# Salvinorin A: A Mini Review of Physical and Chemical Properties Affecting Its Translation from Research to Clinical Applications in Humans

**Published:** 2014

**Authors:** Edward Orton, Renyu Liu

**Affiliations:** 1 Cetazam Therapeutics, 3160 Chestnut Street, Suite 200, Philadelphia PA 19104; 2 Department of Anestheisology and Critical Care, University of Pennsylvania

## Abstract

Salvinorin A is a potent and selective agonist of kappa opioid receptors in the brain. Recent studies in several animal models have revealed that Salvinorin A has anti-addiction, anti-depression properties and exhibits pronounced neuroprotective effects against hypoxia/ischemia induced brain damage, and have raised interest in potential clinical applications in several acute pathologies involving oxygen deficiency in the brain. This review focuses on the chemical and physical properties of Salvinorin A and their impact on development of a rational formulation to enable its translation from a research compound to a novel therapeutic agent.

## INTRODUCTION

**Figure 1:**
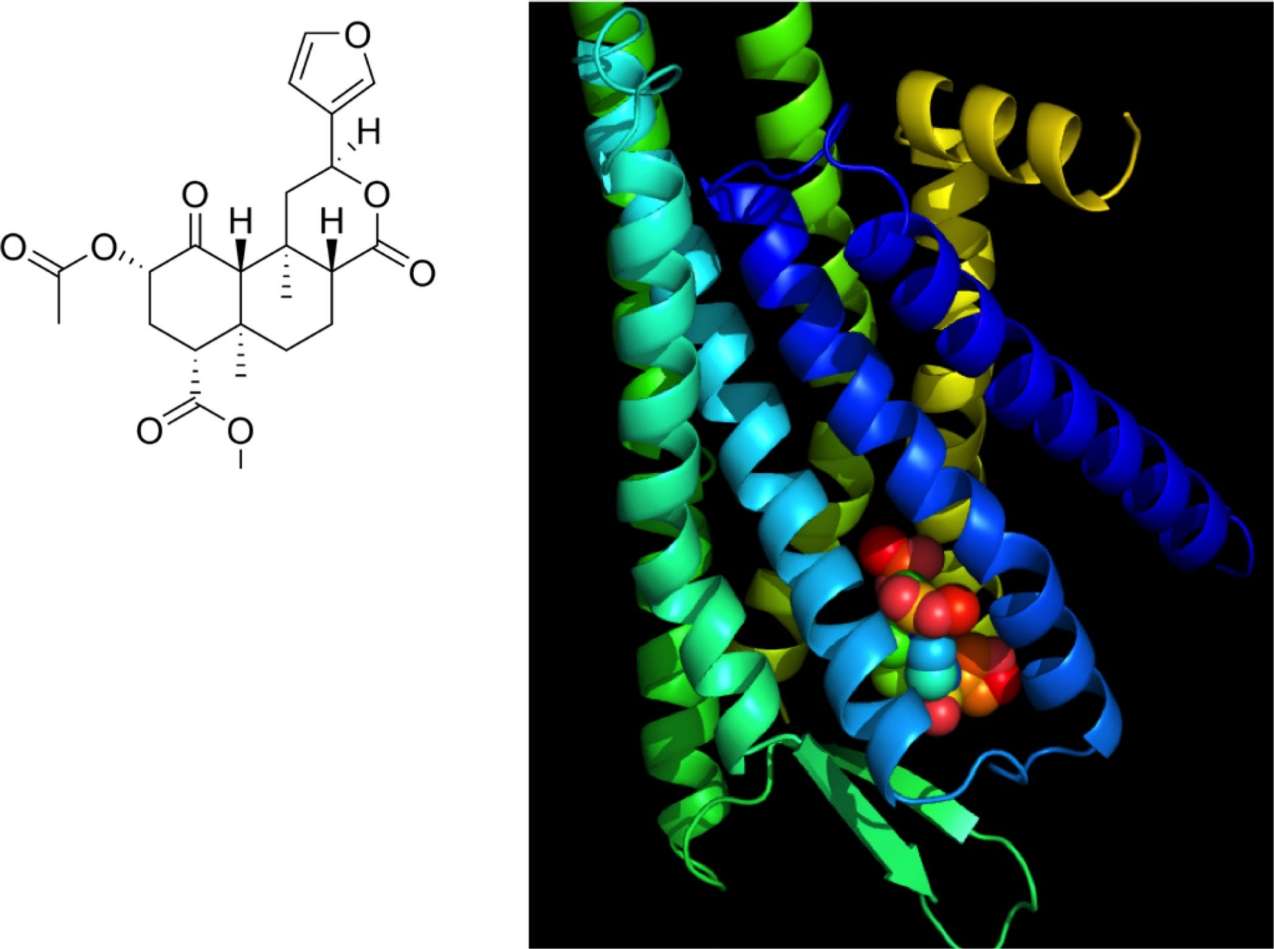
**a)** Molecular structure of Salvinorin A. **b)** Salvinorin A occupies the binding site in the kappa opioid receptor. The binding site prediction was carried out using DockingServer20 as previously described[21]. The coordinates of the crystal structure of the kappa receptor were obtained from the protein data bank (PDB) with access code of 4DJH[22]. The image was generated using PyMOL (Version 1.5.0.4, Schrodinger LLC, New York, NY).

Salvinorin A (SA) is a psychoactive neoclerodane diterpenoid (Figure 1a) isolated from the plant Salvia divinorin, a member of the Sage family that occurs naturally in Mexico[1]. As a recreational drug, SA is typically either smoked or the leaves are chewed to allow absorption of the active drug through the mucous membranes of the oral cavity and giving rise to short-lived intense hallucinations, antinociception, sedation and dysphoria. A highly potent drug, Salvinorin A shows central nervous system (CNS) activity at doses of 200–500 μg.[1–3] After PO dosing in humans, SA is rapidly degraded by enzymes in the intestinal tract resulting in complete loss of exposure[1]. Similarly, the efficacy of SA as a hallucinogen via buccal or sub-lingual routes is diminished in saliva. Salvinorin A has also been shown to be a substrate for P-glycoprotein efflux transporters that are highly expressed in the blood brain barrier, and this contributes to the short cerebral residence time of SA[4].

Since the discovery of SA as the only known naturally-occurring, non-nitrogenous kappa opioid receptor (KOR) agonist (Figure 1b),[5] research leading to clinical use of SA as a potential novel medication for addiction,[6, 6, 7] depression management,[8] and as a neuroprotective agent for neonatal hypoxia/ischemia, as well as brain injury due to oxygen insufficiency arising from cardiac arrest and stroke has been described in recent literature[9]. Specific cerebrovasodilation effects were observed in a newborn pig model which indicated that Salvinorin A is a fast-onset, short-lived agent that may find therapeutic applications in a number of acute brain pathologies accompanied by vasoconstrictive events[10].

In this review we discuss the chemical and physical properties of Salvinorin A and their roles in the process of defining a rational translation pathway for formulating the drug substance into a clinically deliverable dosage form for acute medical situations.

### Chemical and Physical Properties

Physical and chemical properties of SA (see Table 1) reveal a compound with subtle and potentially complex issues from a formulation perspective.

Lacking ionizable functional groups, Salvinorin A cannot form soluble salts. SA has eight hydrogen-bond acceptor sites, all oxygen atoms, and no hydrogen-bond donor groups. It would thus appear likely that its crystal lattice should comprise only weak non-bonded interactions between adjacent molecules and a resultant diminished lattice energy reflected in a low melting point[11]. In fact, SA has a rather high melting point range reported as 238–240 °C. An x-ray crystal structure of SA revealed that crystallization from aqueous organic solvents (acetone, methanol) yields SA as a stoichiometric hydrate with one molecule of water per three molecules of Salvinorin A[12]. Unfortunately, the x-ray structure did not resolve the positions of the water hydrogen atoms, so a detailed picture of the hydrogen-bonding linking SA molecules into the crystal lattice remains unclear. The existence of Salvinorin A as a high melting crystalline hydrate can be expected to influence its solubility behavior in organic and aqueous solvents Poor solubility will likely limit formulation approaches accessible for translation of the drug into a clinically applicable medicine.

The chemical stability of Salvinorin A has not been examined in published literature to date. Nonetheless, any clinically relevant formulation will need to circumvent hydrolytic conditions that may affect the ester and lactone moieties essential to the activity of SA at the KOR receptors. For example, hydrolysis of the acetate in SA produces the well-known derivative Salvinorin B which lacks KOR affinity[13]. Further, the presence of three epimerizable carbons in SA raises concerns over the stereochemical integrity of the drug substance. Chromatographic and spectroscopic analytical methods adequate to detect epimers of SA have been reported[14]. In addition, direct photochemical degradation of SA is not a concern since its ultraviolet absorption onset (due to the furan chromophore) is less than 220 nm.

### Rational Formulation Approaches for Salvinorin A

In view of Salvinorin A’s susceptibility to enzymatic degradation as well as being a substrate for P-glycoprotein efflux, the PO route of administration is clearly not viable. Not surprisingly due to the poor aqueous solubility of Salvinorin A, intravenous (IV) formulations described in numerous in vivo studies over the past 15 years have largely employed pharmaceutically unacceptable solvents (dimethylsulfoxide, methanol and acetone). IV formulations using moderately high percentages of ethanol and propylene glycol[14] as co-solvent may be acceptable for acute clinical situations[15]. The IV route permits some control over duration of the therapeutic dosing level, a matter of concern for the psychoactive side effects of Salvinorin A. An IV nanocrystal formulation of SA may be an alternative to the co-solvent approach, especially when protracted therapeutic brain levels of the neuroprotectant drug are desired.

Despite the poor apparent bioavailability of Salvinorin A when dosed via the oral cavity by recreational drug users,[4] for acute dosing this remains a potential route of administration. Ready access to the oral cavity is a clear advantage for buccal delivery, as is the ease of administration even in the absence of medical personnel. In principle, high doses of SA could be delivered through the oral mucosa using mucoadhesive gels or patches in combination with penetration enhancers to achieve therapeutic levels of SA in the brain. Other advances in buccal delivery systems have been described recently[16]. Suspensions of Salvinorin A as micronized or nanocrystalline particles would be compatible with such delivery systems and act, in effect, to provide prolonged exposure of SA to the brain.

Intranasal administration for direct to brain drug delivery offers several important advantages over other administration routes: rapid onset of therapeutic activity; bypassing the blood brain barrier; avoiding hepatic first pass metabolism[17]. In the case of Salvinorin A for acute neuroprotective therapy a multitude of dosing devices are currently in use (or in development) that can enable initiation of therapy by non-medical persons. The existence of a plurality of intranasal delivery systems that can deliver SA suspension formulations make this an attractive approach. A clear shortcoming of intranasal delivery may be anticipated in patient to patient variability of response due to a combination of intrinsic (e.g. anatomical features) and extrinsic (e.g. accuracy of dosage delivery to site) factors.

Inhalation of Salvinorin A via smoking or vaporization is well known to provide very rapid brain uptake and intense psychotropic effects[18, 19]. Inhalation of medicines is a well-developed field and a large variety of delivery devices and formulations are currently marketed. However, in the context of acute treatment of the unconscious and/or unresponsive patient, inhaled dosage delivery appears problematic at least outside the hospital setting.

## CONCLUSIONS

In this review we have examined the chemical and physical properties of Salvinorin A, a potential novel drug for neurological diseases, to define viable approaches to developing formulations that can be translated to clinically relevant dosage forms for treatment of acute onset illnesses outside a hospital setting. Salvinorin A exists as a stable stoichiometric hydrate and exhibits very poor solubility in aqueous vehicles. Intravenous administration may be achievable with elevated co-solvent vehicles or nanocrystalline suspensions, although its utility in the absence of medical staff is doubtful. Dosage forms targeting the oral mucosa appear worthy of further investigation: while the bioavailability is low, the ability to readily deliver high doses to the oral cavity (a highly accessible site) may allow maintenance of therapeutic drug levels in the brain. Intranasal drug delivery affords rapid, direct access of the drug to the brain bypassing the hepatic circulation. A multitude of choices for formulation and delivery devices exist, but patient to patient variability to this route of drug delivery remains uncertain particularly for administration by non-medical persons. Preferred among recreational drug users, delivery of Salvinorin A via inhalation offers clear advantages in terms of rapidly reaching therapeutic drug levels in the brain. For the purposes of acute treatment of an unresponsive patient, inhalation delivery may not be suitable.

## Figures and Tables

**Table I:** Physical properties of Salvinorin A.

Property	Value	Comments
Molecular weight	432.464 g/mol	Anhydrous
Molecular formula	C23H28O8	Anhydrous
Crystal hydrate molecular formula	C23H28O8 .1/3 H2O	Trienhydrate crystal form
Melting point	238–240 °C	Trienhydrate crystal form
Calculated LogP	2.49	ChemAxon/Marvin
pKa	None	Non-ionizable
Chiral centers	7	3 epimerizable
Optical rotation	−41 deg C at 25 °C	c = 1 in CHCl3
Water content	1.4% (Karl Fischer)	Trienhydrate crystal form
